# Tau-dependent HDAC1 nuclear reduction is associated with altered VGluT1 expression

**DOI:** 10.3389/fcell.2023.1151223

**Published:** 2023-05-17

**Authors:** Giacomo Siano, Giuseppe Madaro, Maria Claudia Caiazza, Awatef Allouch, Martina Varisco, Marianna Mignanelli, Antonino Cattaneo, Cristina Di Primio

**Affiliations:** ^1^ Laboratorio di Biologia Bio@SNS, Scuola Normale Superiore di Pisa, Pisa, Italy; ^2^ Sheffield Institute for Translational Neuroscience (SITraN), University of Sheffield, Sheffield, United Kingdom; ^3^ Department of Physiology, Anatomy and Genetics, Oxford Parkinson’s Disease Centre, University of Oxford, Oxford, United Kingdom; ^4^ Cell Death, Immunity and Therapeutic Innovation Team, Gustave Roussy Cancer Campus, Villejuif, France; ^5^ Rita Levi-Montalcini European Brain Research Institute, Rome, Italy; ^6^ Institute of Neuroscience, Italian National Research Council (CNR), Pisa, Italy

**Keywords:** Tau, HDAC1 histone deacetylase, VGluT1, tauopathies, nuclear HDAC1 reduction

## Abstract

During AD pathology, Tau protein levels progressively increase from early pathological stages. Tau altered expression causes an unbalance of Tau subcellular localization in the cytosol and in the nuclear compartment leading to synaptic dysfunction, neuronal cell death and neurodegeneration as a consequence. Due to the relevant role of epigenetic remodellers in synaptic activity in physiology and in neurodegeneration, in particular of TRIM28 and HDAC1, we investigated the relationship between Tau and these epigenetic factors. By molecular, imaging and biochemical approaches, here we demonstrate that Tau altered expression in the neuronal cell line SH-SY5y does not alter TRIM28 and HDAC1 expression but it induces a subcellular reduction of HDAC1 in the nuclear compartment. Remarkably, HDAC1 reduced activity modulates the expression of synaptic genes in a way comparable to that observed by Tau increased levels. These results support a competitive relationship between Tau levels and HDAC1 subcellular localization and nuclear activity, indicating a possible mechanism mediating the alternative role of Tau in the pathological alteration of synaptic genes expression.

## Introduction

Tau is a microtubule (MT) associated protein whose primarily described function is to stabilize the cytoskeleton and to regulate the vesicle transport in neurons. Progressive Tau destabilization from MTs and cytotoxic aggregation characterize a group of neurodegenerative diseases called Tauopathies (Wang and Mandelkow, 2016).

An extensive body of evidence demonstrated the crucial role of Tau aggregation in tauopathies.

Tau aggregation is most frequently discussed in the context of a gain-of-toxic-function. It has become clear that only focusing on the gain-of-toxic effect of Tau aggregates might lead us to overlook certain aspects of the pathological phenotype that cannot be explained by protein aggregation only. Thus, aggregation can also lead to a loss-of-function, be it the canonical function of tau as a microtubule stabilizer, or of other physiological functions of tau. Therefore, recently, a surge of interest in Tau physiology has been observed and how its alterations might lead to cytotoxicity and neuronal cell death (Arendt et al., 2016; Sotiropoulos et al., 2017; Siano et al., 2021; Song et al., 2021). Tau has been linked to a number of so-called “non-canonical” functions including gene regulation and DNA protection in the nucleus, synaptic transmission at the postsynaptic terminal and mitochondrial dynamics. Remarkably, these non-canonical functions are significantly altered during neurodegeneration when Tau is destabilized and displaced from MTs and forms oligomers, suggesting that Tau already contributes to cell damage at early stages of the disease (Sotiropoulos et al., 2017; Siano et al., 2021; Tracy et al., 2022).

We described that the relocation and accumulation of Tau protein in the nucleus in the early Alzheimer’s disease (AD) stages cause changes in the expression of disease-related genes, among these, the gene encoding the glutamate transporter VGluT1 and other genes involved in glutamate synaptic transmission. With disease progression, Tau-dependent gene expression is impaired by aggregation indicating that aggregation leads to nuclear Tau loss of function (Siano et al., 2019b; Siano et al., 2020).

The mechanisms mediating this function are still unclear. However, it was demonstrated that Tau interacts with Tripartite Motif Containing 28 (TRIM28), a scaffold protein that forms big complexes with transcription factors and epigenetic remodellers such as DNA methyl transferases (DNMTs), Histone deacetylases (HDACs) and Histone acetyltransferases (HATs). The interaction between TRIM28 and Tau is significantly associated to pathological transport of Tau into the nucleus and with chromatin changes, thus suggesting Tau involvement in as yet undescribed nuclear mechanisms (Iyengar and Farnham, 2011; Rousseaux et al., 2016). Moreover, an increasing body of evidence confirms a relevant epigenetic alteration during AD even associated to Tau pathological forms (Frost et al., 2014; Mano et al., 2017; Gil et al., 2021).

In order to gain more insight into the mechanisms mediating Tau-mediated changes in gene expression, we investigated the relationship between tau, TRIM28 and HDACs, using the expression of the glutamate transporter VGluT1 as an experimental read-out of Tau-dependent gene expression. Here we describe a mechanism that involves a competitive relationship by HDAC1 on Tau-TRIM28 interaction. This competition leads to the reduction of HDAC1 in the nucleus resulting in the alteration of disease-related genes. This result supports the hypothesis that HDAC1 displacement from the nucleus mediated by Tau upregulation could be a mechanism involved in disease-related gene expression alterations observed in tauopathies.

## Materials and methods

### Cell culture and treatments

The SH-SY5Y human neuroblastoma line is widely used for studying Tau physiology and pathology in the context of AD and tauopathies (Teppola et al., 2016; Riegerová et al., 2021; Strother et al., 2021; Bell and Zempel, 2022). SH-SY5Y cells were cultured in Dulbecco’s Modified Eagle Medium/Nutrient Mixture F12 (DMEM/F-12 Gibco) supplemented with 10% Fetal Bovine Serum (FBS, Euroclone), L-Glutamine (Euroclone), 100 U/mL Penicillin and 100 μg/mL Streptomycin (Euroclone). Differentiation was performed as previously described (Siano et al., 2019a). SH-SY5Y cells were treated with 10 μΜ retinoic acid (RA) (Sigma-Aldrich) for 5 days in complete medium followed by 50 ng/mL Brain-derived neurotrophic factor (BDNF; Alomone) for 3 days in DMEM/F-12 supplemented with L-Glutamine. Cell transfection has been performed by Lipofectamine 2000 the day after plating according to manufacturer’s protocol. For HDACs inhibition, differentiated SH-SY5Y have been treated with 800 nM Trichostatin A (TSA) (Sigma) for 16 h (h). HDAC1 knockdown experiment was performed by the specific HDAC1 esiRNA (Sigma-Aldrich ID HU-02584-1). HDAC1 esiRNA has been transfected by Lipofectamine 2000 according to manufacturer’s protocol.

### HDAC activity assay

Total HDACs activity was assayed by means of Epigenase™ HDAC Activity/Inhibition Direct Assay Kit (Epigenetek) according to the manufacturer’ instructions. Briefly, an acetylated histone HDAC substrate is stably coated onto the microplate wells. Total protein extract is collected and added to the plate. Active HDACs bind to the substrate and removes acetyl groups from the substrate. The enzymatic reaction products are detected by a specific antibody. OD signal of deacetylated products is measured by a microplate spectrophotometer at 450 nm.

### Co-immunoprecipitation

For co-immunoprecipitation experiments, total cell extracts were prepared in ice-cold RIPA buffer (150 mM NaCl RIPA) supplemented with protease and phosphatase inhibitors (Roche). The lysate was quantified using Bradford assay (Thermo Fisher Scientific) and 2 mg of protein were incubated with 2 µg of primary antibodyTau13 overnight at 4°C. 20 µL of agarose resin conjugated protein G (Santa Cruz) or magnetic beads conjugated protein G (BioRad) were incubated for 2 h at 4°C. Samples were washed in ice-cold RIPA. Samples have been processed for WB as described below.

### Western blot

For Western blot experiments, 4 × 10^5^ cells per well were plated in 6-well plates. Total cell extracts were obtained by suspending cell pellets in 100 µL of cell lysis buffer (Tris-HCl 20 mM pH 8.1, NaCl 20 mM, glycerol 10%, NP-40 1%, EDTA 10 mM, supplemented with protease and phosphatase inhibitors) followed by incubation for 30 min on ice. Cell lysates were centrifuged at 16,000 rcf for 15 min at 4°C. The supernatant was collected and quantified by Bradford method. Subcellular fractionation was performed using the Subcellular Protein Fractionation Kit for Cultured Cells (ThermoFisher Scientific) as previously described (Siano et al., 2019a). 20 μg of protein were mixed with 4X Laemmli Buffer and denatured at 95°C for 5 min. Samples were separated by 10% Tris-Glycine SDS-PAGE (Bio-Rad) and electro-blotted onto nitrocellulose membranes Hybond-C-Extra (Amersham Biosciences). Membranes were blocked with blocking solution (5% w/v skimmed milk powder in TBST), hybridized with the primary antibodies O/N at 4°C, washed with TBST (20 mM Tris, 150 mM NaCl, 0.1% Tween 20, pH 7.4–7.6) and then incubated with HRP-conjugated secondary antibodies for 1 h at RT. Antibodies were diluted in TBST with 1% w/v skimmed milk powder. Membranes were developed using chemiluminescent HRP substrates: GE Healthcare (Life Sciences), Immobilon (Millipore) and SuperSignal (Thermo Fisher Scientific) depending on protein expression level and antibody specificity. Tau13 (sc-21796) Santa Cruz 1:1000; VGluT1 (ab77822) Sigma-Aldrich 1:1000; TRIM28 (# A300-274A) Bethyl 1:1000; HDAC1 (ab53091) Abcam 1:1000; GAPDH (10R-G109a) Fitzgerald 1:15000; HRP-conjugated anti-mouse Santa Cruz Biotechnology 1:1000; HRP-conjugated anti-rabbit Santa Cruz Biotechnology 1:1000. Molecular weights: TRIM28 = 110 kD; HDAC1 = 60 kD; Tau = 50 kD; VGluT1 = 60/70; GAPDH = 30 kD, H2B = 28 kD. All blots have been normalized to the internal control and the housekeeping proteins.

### Immunofluorescence and image acquisition

Cells were plated on chamber slides (LabTek) and fixed 24 h post transfection with ice-cold methanol for 4 min. After fixation cells were permeabilized with 0.1% Triton X-100 in PBS and blocked for 30 min with the blocking solution (PBS + Tween 0.1%+ BSA 1% fresh). Primary antibody incubation has been done in blocking solution for 1 h at room temperature or overnight at 4°C.

Immunofluorescence images were acquired with the confocal laser-scanning microscope TCS SL (Leica Microsystems) equipped with Leica Application Suite (LAS) X software. All frames were captured by means of a 63X/1.4 NA HCX PL APO oil immersion objective, a format size of ×512 512 pixel and a sequential scan procedure. An Argon laser was used for Alexa Fluor 488 (*λ* = 488 nm), a He-Ne laser for Alexa Fluor 633 (*λ* = 633 nm).

### Data analysis and statistics

Western blot and immunofluorescence images have been analysed by ImageJ software. For Western blot analyses, non-parametric Mann-Whitney test or Kruskal–Wallis test followed by pairwise Mann-Whitney test were used. In fluorescence intensity analysis, Region Of Interest (ROI) of HDAC1 positive nuclei have been considered. ROI has been selected by merging of channels and bright-field. Nuclear mean fluorescence intensity has been measured and statistical significance was determined by one-way analysis of variance ANOVA followed by Tukey multiple comparisons test. For all the experiments, at least three biological replicates have been performed. In bar-plots, values are expressed as the mean (bar) and SEM (whiskers). Significance is indicated as *p < 0.05, **p < 0.01 and ***p < 0.001.

## Results

### Tau expression does not affect TRIM28 expression and localization

We demonstrated that Tau destabilization from MTs and its increased amount as a soluble protein not associated to MTs, typically occurring during AD progression, alters the expression of synaptic genes associated with AD pathology (Wang and Mandelkow, 2016; Han et al., 2017; Siano et al., 2019b). In addition, it has been previously observed that in AD brains Tau interacts with TRIM28 which mediates its translocation into the nuclear compartment leading to chromatin reorganization and cell damage (Rousseaux et al., 2016).

**FIGURE 1:**
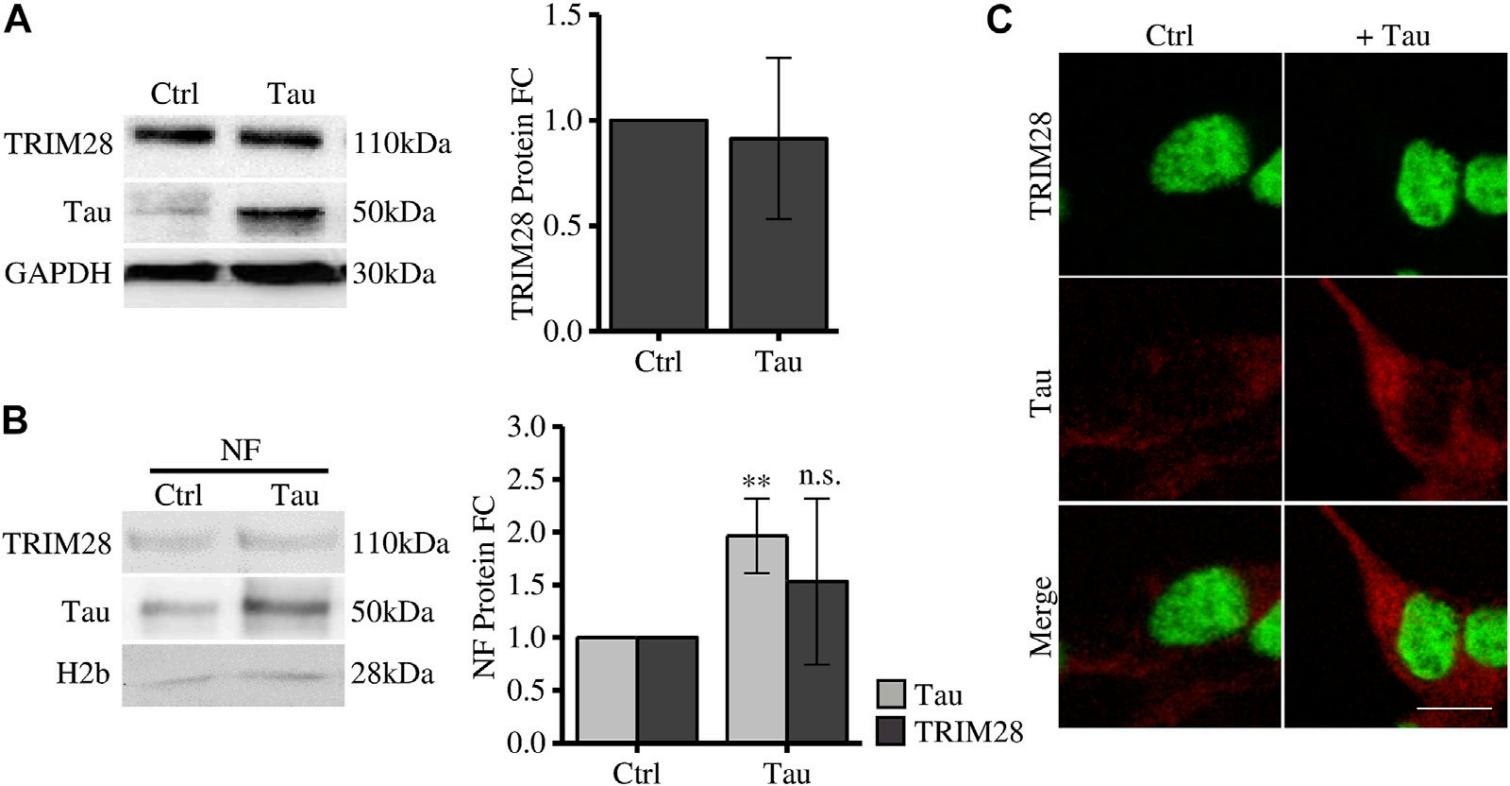
Tau expression does not affect TRIM28 expression and localization. **(A)** Western blot and relative quantification (FC = fold change) of differentiated SH-SY5Y overexpressing Tau (Tau) and control cells (Ctrl). **(B)** Subcellular fractionation of Tau-overexpressing SH-SY5Y and control cells. TRIM28 and Tau analysis by Western blot and relative quantification. NF: nuclear fraction. **(C)** Confocal microscopy immunofluorescence of TRIM28 (green), Tau (red) and in Tau-overexpressing SH-SY5Y and control cells (Scale bar = 10 µm). Molecular weights: TRIM28 = 110 kDa; Tau = 50 kDa; GAPDH = 30 kDa, H2b = 28 kDa **p < 0.01.

To elucidate the Tau-dependent mechanism altering disease-related genes expression, we investigated the interplay between Tau and TRIM28 in Tau overexpressing SH-SY5Y cells. The expression level of TRIM28 is not altered after increasing Tau expression (Figure 1A). By subcellular fractionation, we observed a significant increase of Tau in the nuclear compartment, as expected. However, TRIM28 nuclear accumulation was not modified by Tau overexpression (Figure 1B). These data were further confirmed by immunofluorescence experiments (Figure 1C).

Altogether, these results indicate that, while TRIM28 drives nuclear Tau translocation in pathological conditions (Rousseaux et al., 2016), Tau does not alter TRIM28 expression and subcellular localization.

### Tau dictates HDAC1 subcellular localization

**FIGURE 2:**
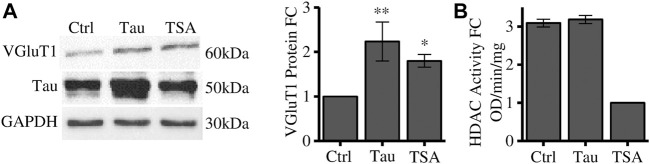
HDACs inhibition alters VGluT1 expression levels. **(A)** Western blot analysis of differentiated SH-SY5Y cells overexpressing Tau or treated with TSA and relative quantification. The levels of VGluT1 have been determined as a paradigmatic Tau-modulated reference gene. **(B)** HDAC colorimetric assay of control cells, Tau overexpressing cells and TSA-treated cells. Molecular weights: Tau = 50 kDa; VGluT1 = 60/70 kDa; GAPDH = 30 kDa, *p < 0.05; **p < 0.01.

TRIM28 is a scaffold protein that mediates the interaction with several proteins associated to chromatin remodelling and gene expression (Iyengar and Farnham, 2011). Among these proteins we focused on HDACs which regulate heterochromatin formation. Cells have been treated with Trichostatin A (TSA), a pan-HDAC inhibitor, and VGluT1 expression has been employed as a readout of the Tau-dependent gene expression regulation. We observed that HDACs bulk inhibition in naïve cells leads to a significant increase in VGluT1 levels comparable to its increase induced by Tau overexpression (Figure 2A). We verified by colorimetric assay that Tau overexpression does not impair HDACs deacetylase activity. Here, TSA was used as positive control of HDACs inhibition (Figure 2B).

These results suggest that HDACs could regulate the expression of disease-related genes such as VGluT1, however, their enzymatic activity is not directly affected by Tau overexpression. To further investigate the possible involvement of Tau in this mechanism we focused on HDAC1. HDAC1 is a key factor for the neuronal activity. Indeed, it is directly associated with the appropriate maintenance of long term potentiation of synapses and with the regulation of synaptic transmission in tauopathies (Patnaik et al., 2021; Grabowska et al., 2022).

**FIGURE 3:**
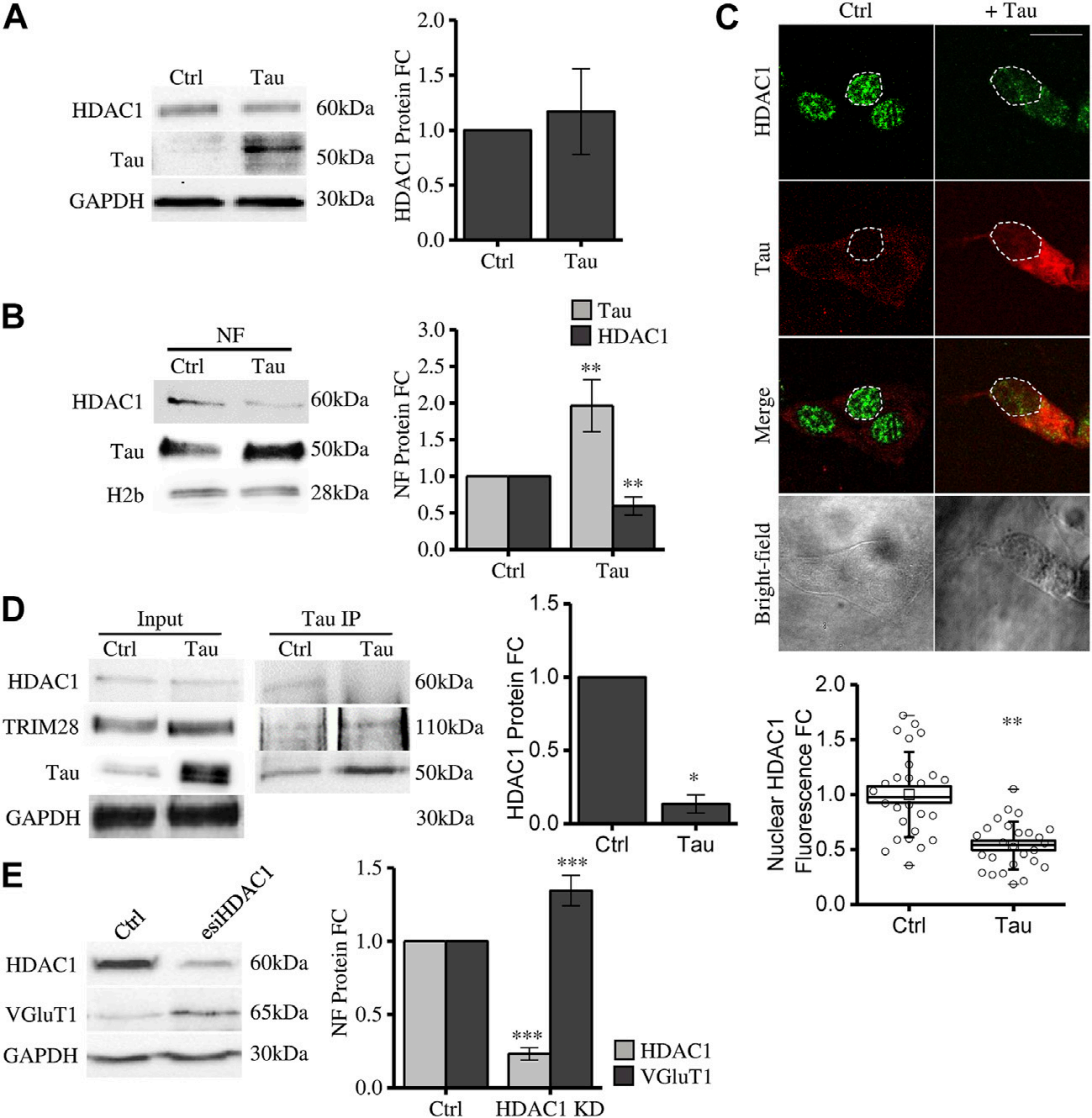
Tau nuclear accumulation causes HDAC1 displacement from the nuclear compartment leading to VGluT1 increase. **(A)** Western blot and relative quantification of differentiated SH-SY5Y cells overexpressing Tau (Tau) and control cells (Ctrl). **(B)** Subcellular fractionation of Tau-overexpressing and control cells. HDAC1 and Tau analysis by Western blot and relative quantification in the nuclear fraction (NF). **(C)** Immunofluorescence of Tau-overexpressing SH-SY5Y and control cells. HDAC1 (green); Tau (red); bright-field (grey) (Scale bar = 10 µm). Quantification of HDAC1 fluorescence fold change in arbitrary units (a.u.) (*n* = 26). **(D)** Co-immunoprecipitation experiment and relative quantification by anti-Tau antibody and detection of TRIM28 and HDAC1. **(E)** Western blot of SH-SY5Y cells knock down for HDAC1 and VGluT1 analysis. Molecular weights: TRIM28 = 110 kDa; HDAC1 = 60 kDa; Tau = 50 kDa; VGluT1 = 60/70 kDa; GAPDH = 30 kDa, H2b = 28 kDa *p < 0.05; **p < 0.01; ***p < 0.001.

We observed that Tau overexpression does not alter total HDAC1 expression level (Figure 3A). However, HDAC1 protein is significantly reduced in the purified nuclear compartment (Figure 3B). This result is further confirmed by confocal microscopy immunofluorescence experiments (Figure 3C). These data show that increasing nuclear Tau by Tau overexpression (Siano et al., 2019b) somehow decreases the pool of HDAC1 in the nucleus, suggesting a competitive interplay between Tau and HDAC1 in the nuclear compartment. To investigate this hypothesis, we performed a Co-immunoprecipitation (coIP) experiment. In control conditions both endogenous Tau and HDAC1 take part of the TRIM28 complex. Upon Tau expression, while the interaction between Tau and TRIM28 was preserved, HDAC1 signal was reduced (Figure 3D). This evidence supports the hypothesis that the increased availability of nuclear Tau might compete with HDAC1 in the formation of the complex with TRIM28.

To elucidate whether HDAC1 nuclear displacement might have a role in Tau-dependent gene expression, we checked the expression of VGluT1 in HDAC1 knockdown (KD) cells (see Methods). We observed that HDAC1 KD is associated with a significant increase in VGluT1 expression (Figure 3E). This result supports the hypothesis that HDAC1 displacement from the nucleus mediated by Tau upregulation could be a mechanism involved in disease-related gene expression alterations observed in tauopathies.

## Discussion

In Alzheimer’s disease and tauopathies, the neuronal protein Tau is progressively detached from microtubules, mostly due to its hyperphosphorylation and forms aggregates that lead to neuronal damage and cell death (Wang and Mandelkow, 2016). Several studies in the last 10 years demonstrated additional non-canonical functions to its well-established microtubule-regulating role; these functions are currently under investigation to understand the pathology and prevent or reduce neuronal damage (Guo et al., 2017; Sotiropoulos et al., 2017; Deture and Dickson, 2019; Siano et al., 2021). Tau aggregation is only one of the aspects linked to neurodegeneration. Tau loss of function in MTs dynamics or the alternative non canonical functions might be a highly relevant disease mechanism.

We recently demonstrated that Tau destabilization from MTs and its increased expression as a soluble MT-unbound protein, occurring at early stages of the disease, cause a significant increase in Tau nuclear levels, changing the expression of genes involved in synaptic transmission and leading to toxic hyperexcitability (Siano et al., 2019b). Accordingly, neuronal hyperexcitability, associated also with VGluT1 increase, has been identified as a hallmark of early AD pathology (Ghatak et al., 2019; Siano et al., 2020; Taipala et al., 2022). The molecular mechanisms mediating this effect are still elusive. Several evidences suggest an interplay between Tau and chromatin structure since Tau can directly bind DNA and histones and can induce transposon instability (Wei et al., 2008; Benhelli-Mokrani et al., 2018; Guo et al., 2018; Gil et al., 2021; Rico et al., 2021). However, it is still unclear if these aspects may cause alterations of the expression of synaptic-related genes.

Searching for proteins that might interact with the nuclear pool of Tau, we focussed the attention on TRIM28, a known interactor of Tau, and a key nuclear protein involved in heterochromatin regulation by binding transcription factors and chromatin remodellers (Iyengar and Farnham, 2011; Rousseaux et al., 2016). We investigated here if this interaction might play a role in Tau-dependent gene expression changes. We previously used a human neuroblastoma cell line overexpressing Tau to describe how nuclear Tau modulates the expression of genes that are relevant for the early stages of AD (Siano et al., 2019b). Tau destabilization from MTs or its overexpression cause a significant increase of Tau protein both in the soluble and in the nuclear fractions in different cellular types and in cancer and primary neurons (Benhelli-Mokrani et al., 2018; Siano et al., 2019a; Siano et al., 2019b; Rico et al., 2021; Siano et al., 2021). Here, we used this cellular model to investigate the interplay between Tau, TRIM28 and HDAC1. We confirmed also in this cellular model the established interaction between Tau and TRIM28 by co-IP and we did not observe any significant Tau-induced change in TRIM28 expression. In addition, Tau does not affect TRIM28 nuclear localization.

Since Tau directly binds TRIM28 without affecting its expression nor its localization, we investigated if Tau is able to affect other TRIM28 interactors. Among such co-factors, TRIM28 binds some HDACs, nuclear enzymes that reduce chromatin acetylation leading to heterochromatin formation and regulating gene expression (Iyengar and Farnham, 2011). To check whether HDAC activity could affect the expression of genes that according to our previous reports can be altered by Tau (Siano et al., 2021), we treated cells with the pan-HDAC inhibitor TSA and assessed VGluT1 amount as a Tau-dependent reporter gene. The HDAC inhibition caused a significant increase of VGluT1 levels, comparable with that observed after Tau higher expression. This result suggests that HDACs may be involved in the altered expression of VGluT1. After verifying that Tau did not affect HDAC activity, we checked whether it could alter the properties of a specific nuclear HDAC.

Although it is well-known that Tau interacts with HDAC6 in the cytoplasm (Ding et al., 2008), to investigate the Tau-dependent gene expression mechanism we focused on nuclear HDACs. In particular HDAC1 has been reported to participate in synaptic transmission regulation mainly associated to neuronal excitability and to be necessary for synaptic plasticity maintenance (Akhtar et al., 2009; Han et al., 2021; Grabowska et al., 2022). Remarkably, HDAC1 is also one of the interactors of TRIM28 (Allouch et al., 2011; Iyengar and Farnham, 2011). Due to this evidence supporting HDAC1 relevant role in excitatory transmission specifically in the nuclear compartment, we focused on the interplay between Tau expression and HDAC1 and observed that Tau does not alter HDAC1 expression. However, surprisingly, HDAC1 nuclear levels are reduced concomitantly with nuclear Tau increase. These results indicate that Tau increased amount leads to a reduction of nuclear HDAC1, without altering its enzymatic activity, as assessed from whole cell extracts. This observed Tau-induced reduction of HDAC1 in the nucleus, in turn, prevents HDAC1 from exerting its physiological function in the nuclear compartment. Indeed, consistently with this prediction, HDAC1 knockdown causes a significant increase of VGluT1 expression.

**FIGURE 4:**
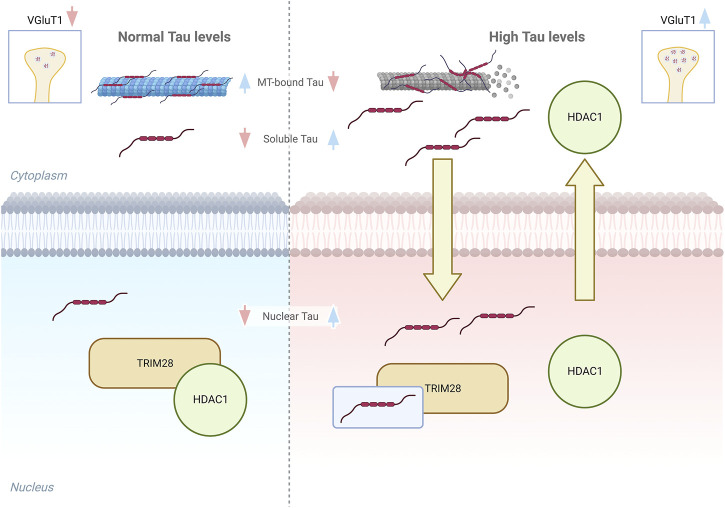
Graphical model of Tau interplay with TRIM28/HDAC1 complex. Left panel, in healthy conditions the amount of soluble cytoplasmic and nuclear Tau is low and do not alter the interaction between TRIM28 and HDAC1. Right panel, higher soluble Tau levels lead to an increase of soluble and nuclear Tau and HDAC1 displacement from TRIM28, with its consequent export from the nucleus.

Altogether these results support a potential mechanism mediating Tau-dependent regulation of the synapses-related genes in early AD stages. Accordingly, we hypothesize that when soluble Tau proteins increase in both cytoplasm and nucleus, due to Tau displacement from microtubules and its accumulation (Wang and Mandelkow, 2016; Han et al., 2017), the interaction with TRIM28 is increased, impairing HDAC1 interaction with the remodeller complex based on TRIM28 scaffold. This competitive effect would cause HDAC1 reduction in the nucleus thus preventing its nuclear transcriptional repression functions. Indeed HDAC1 targets histones and several transcription factors that might be involved in Tau-dependent VGluT1 increased expression (Nalawansha et al., 2018) (Figure 4). HDAC1 is strongly associated with the correct functioning of synaptic transmission and axonal damage and its interplay with Tau suggests that the Tau-dependent reduction of HDAC1 availability in the nucleus might also affect neuronal homeostasis (Zhu et al., 2017; Grabowska et al., 2022). Remarkably, HDAC1 nuclear export has been already described in neurons under pathological stimuli and in multiple sclerosis brains and its export is linked to axonal damage and mitochondrial transport impairment (Kim et al., 2010; Zhu et al., 2017). Intriguingly, these events can be also observed in AD, suggesting that alterations of the pathological Tau-HDAC1 interplay could be involved in several neurodegenerative processes. In addition to this, post-translational modifications of Tau protein modulate its stability, subcellular localization, and function. Acetylation of Tau, mediated by specific enzymes or its intrinsic enzymatic activity, may directly or indirectly influence Tau functions by altering its subcellular localization and subsequent interaction with nuclear co-factors (Cohen et al., 2013; Wang and Mandelkow, 2016). The observed Tau-dependent relocalization of HDAC1 may be attributed to altered acetylation of Tau. Further experimentation is required to elucidate this phenomenon and to determine the potential involvement of other nuclear HDACs, such as HDAC2. Additionally, the involvement of cytoplasmic enzymes, such as HDAC6 which interacts with Tau (Ding et al., 2008), in this specific Tau-dependent alteration cannot be excluded.

In conclusion, this evidence indicates for the first time a novel pathological mechanism mediated by Tau that could partially explain the deregulation of synaptic genes in early AD conditions. The results identify the molecular triad of Tau-TRIM28-HDAC1 as a key target for the development of Tau-modulating therapeutic approaches. Further studies in cellular and in tauopathy mouse models are needed to elucidate the precise competitive mechanisms causing the Tau-dependent reduction of the nuclear pool of HDAC1 and to identify the affected dementia-related genes that could induce or enhance the neuronal dysfunction and death. Remarkably, the Tau-TRIM28-HDAC1 triad might represent a potential target to prevent Tau-mediated modulation of nuclear functions and synaptic gene expression alterations, supporting current therapeutic approaches targeting epigenetic modulators preventing the progression of the disease with the identification of more precise molecular target (Heerboth et al., 2014).

## Data Availability

The raw data supporting the conclusion of this article will be made available by the authors, without undue reservation.
